# Nurse-led care versus physician-led care in the management of rheumatoid arthritis and psoriatic arthritis (StaerkeR): study protocol for a multi-center randomized controlled trial

**DOI:** 10.1186/s13063-019-3808-3

**Published:** 2019-12-30

**Authors:** Anna Mai, Jürgen Braun, Jens-Peter Reese, Benjamin Westerhoff, Ulrike Trampisch, Renate Klaassen-Mielke, Nina Timmesfeld, Hans J. Trampisch, Dietmar Krause

**Affiliations:** 1 Department of Medical Informatics, Biometry & Epidemiology, Ruhr-University Bochum, 44780 Bochum, Germany; 2 Rheumazentrum Ruhrgebiet, 44652 Herne, Germany; 3 Coordinating Center for Clinical Trials of the Philipps-University of Marburg, 35043 Marburg, Germany; 4 BARMER health insurance, 42285 Wuppertal, Germany

**Keywords:** Rheumatoid arthritis, Rheumatology nurse, Nurse-led care

## Abstract

**Background:**

In Germany, the care of patients with inflammatory arthritis could be improved. Although specialized rheumatology nurses could take over substantial aspects of patient care, this hardly occurs in Germany. Thus, the aim of the study is to examine structured nursing consultation in rheumatology practices.

**Methods/design:**

In total, 800 patients with a stable course of rheumatoid arthritis or psoriatic arthritis in 20 centers in North Rhine–Westphalia and Lower Saxony will be randomized to either nurse-led care or standard care. Participating nurses will study for a special qualification in rheumatology and trial-specific issues. It is hypothesized that nurse-led care is non-inferior to standard care provided by rheumatologists with regard to a reduction of disease activity (DAS28) while it is hypothesized to be superior regarding changes in health-related quality of life (EQ-5D-5L) after 1 year. Secondary outcomes include functional capacity, patient satisfaction with treatment, and resource consumption.

**Discussion:**

Since there is insufficient care of rheumatology patients in Germany, the study may be able to suggest improvements. Nurse-led care has the potential to provide more efficient and effective patient care. This includes a more stringent implementation of the treat-to-target concept, which may lead to a higher percentage of patients reaching their treatment targets, thereby improving patient-related outcomes, such as quality of life, functional capacity, and participation. Additionally, nurse-led care may be highly cost-effective. Finally, this project may form the basis for a sustainable implementation of nurse-led care in standard rheumatology care in Germany.

**Trial registration:**

German Clinical Trials Register, DRKS00015526. Registered on 11 January 2019.

## Background

In Germany, the medical care of patients with inflammatory rheumatic diseases could be improved [1]. Two German population-based surveys showed suboptimal achievement of therapeutic targets [2, 3]. German rheumatologists spend much of their time doing routine work, documentation etc., tasks that might be accomplished by qualified rheumatology nurses without any reduction in the quality of care. Thus, in 2007 a network of German professional rheumatology organizations started offering a specialized qualification for rheumatology nurses to enhance their competence in dealing with rheumatic patients.

In 2012, the European League Against Rheumatism (EULAR) recommended involving nurses to improve patient care in rheumatology [4]. Recommendation 1: “Patients should have access to a nurse for education to improve knowledge of CIA (chronic inflammatory arthritis) and its management throughout the course of their disease.” Recommendation 2: “Patients should have access to nurse consultations in order to experience improved communication, continuity and satisfaction with care.” Recommendation 4: “Nurses should participate in comprehensive disease management to control disease activity, to reduce symptoms and to improve patient-preferred outcomes.” Recommendation 5: “Nurses should identify, assess and address psychosocial issues to minimize the chance of patients’ anxiety and depression.” In recent years, international studies have shown non-inferiority or even superiority of nurse-led care compared with standard care [5, 6].

The participation of nurses in comprehensive disease management is well established in some European countries, e.g. in the Netherlands and the UK [5]. In Germany, this is mostly lacking, although specialized rheumatology nurses could take over substantial aspects of patient care. The aim of this study is to improve the care of patients with inflammatory arthritis through the effective and efficient participation of well-educated specialized rheumatology nurses.

### Main hypotheses

The main hypotheses are that nurse-led care is (1) non-inferior to standard care by rheumatologists with regard to a reduction of disease activity after 1 year and (2) superior with regard to changes in health-related quality of life after 1 year of the intervention (hierarchical test).

## Methods/design

StaerkeR is a multi-center randomized controlled trial with two intervention arms (nurse-led versus standard care) conducted in 20 rheumatology practices or outpatient clinics in North Rhine–Westphalia and Lower Saxony, Germany. Most of these practices are members of RheumaNetz Westfalen-Lippe, a local network of rheumatologists in the eastern part of North Rhine–Westphalia.

Study centers must provide rooms for the specialized nurses and for an external assessor measuring the primary outcome of the study. Participating rheumatologists in these study centers are trained in good clinical practice and experienced in randomized clinical trials.

This project is based on the assumption that a well-qualified rheumatology nurse can take over a substantial part of patient care for predefined tasks. The nurse’s qualifications play a central role. They may already be specialized or may have to become specialized as a rheumatology nurse. The Professional Association of German Rheumatologists (BDRh), the German Rheumatology Society (DGRh), and the Rheumaakademie (a national training center) have developed a training curriculum for rheumatology nurses. In accordance with the guidelines of the German Medical Association (Bundesärztekammer), rheumatology nurses are taught the anatomical and functional basics of the musculoskeletal system, pathology, the basics of pharmacokinetics, and how to assess disease progression.

A rheumatology nurse also has to participate in the study, which includes a refresher and training on how to conduct a joint examination as well as study-specific procedures, e.g. standardization, data collection, and monitoring.

### Participants

The target group for this study are patients with rheumatoid arthritis or psoriatic arthritis. Their general health (including comorbidities) must be stable and disease activity should be low.

### Inclusion and exclusion criteria

Inclusion criteria: stable rheumatoid arthritis or polyarticular psoriatic arthritis with the 28-joint Disease Activity Score (DAS28) < 3.2 (low disease activity) and no current adverse drug reactions.

Exclusion criteria: limited mobility, insufficient knowledge of German, highly active disease, or life-threatening disease.

### Sample size

The sample size was calculated by assuming that changes in the DAS28 scores in the nurse-led care group would not be inferior to those in the standard care group after 1 year of intervention, with a standard deviation of 1.7 and a non-inferiority margin of 0.4 [5]. Using a one-sided *t*-test of non-inferiority (α = 0.025) and setting the power to 90% result in a sample size of 380 patients for each study arm. Assuming a dropout rate of 5% finally results in a sample size of 400 patients per group. According to a British study from 2014, this sample size would be able to demonstrate the superiority of health-related quality of life in the nurse-led care group compared to the standard care group (α = 0.05). Thus, 800 patients will be recruited into the trial.

### Recruitment

As a legal framework, this study is being conducted in cooperation with the health insurance company BARMER using selective contracts for the special nurse-led patient care according to §140a of the fifth Social Security Code (SBG V). To be eligible for the study, patients, thus, must have health insurance from BARMER.

Eight out of the 20 participating study centers were already participating in a selective contract with BARMER regarding the integrated care of patients with inflammatory rheumatic diseases. The involvement of a rheumatology nurse was not part of the contract at that time.

In total, 1140 patients signed the selective contract with the insurer. Of these, approximately 80% had rheumatoid arthritis and 10% had psoriatic arthritis. Analyses showed that most patients could be treated so that they were in a stable phase of their disease, thus making them eligible for the StaerkeR study.

Assuming a participation rate of 50% of those patients who have already signed the selective contract with BARMER and by increasing the number of participating study centers to about 20, recruiting the required sample size of 800 patients in 6 months is feasible.

Physicians will pre-screen all suitable BARMER patients with rheumatoid arthritis or psoriatic arthritis for eligibility. They will invite eligible patients for a visit to the practice to check final eligibility and to provide information about the study. Patients who are eligible and willing to participate in the trial must sign a written informed consent form.

### Interventions

The duration of the intervention is 1 year. All patients will be treated in their rheumatology practice or outpatient clinic, irrespective of group allocation. Adherence to the interventions will be monitored by participation in routine control checks.

#### Experimental intervention: nurse-led patient care

In the nurse-led patient care model, a rheumatology nurse will be the primary contact person for 1 year. Every 3 months, the nurse will perform all necessary assessments and will ask questions about comorbidities and risk factors. Patients may also contact the nurse by phone, fax, app, or email beyond these regular appointments.

Pillars of nurse-led care:

- Structured management of routine examinations: course of the disease, comorbidities, infections, hospitalization, vaccination status, medication, adverse drug reactions, lifestyle (e.g., smoking), incapacity for work, height, weight, blood pressure, Clinical Disease Activity Index (CDAI)
- Management of the treat-to-target principle: The nurse informs the rheumatologist about the CDAI score (CDAI > 10 may lead to an adjustment in therapy).
- Structured management in case of hospitalization or discharge from hospital: The nurse will provide all necessary information to the co-workers in the hospital, especially regarding medication before and after hospitalization.
- Improvement of low-threshold accessibility of the rheumatology practice via direct contact with the nurse by telephone, fax, email, or app.
- Annual update of the vaccination plan in coordination with the general practitioner.

To standardize nurse-led care, the nurses have been given a checklist to follow during patient examinations and consultations. The operating plans define the important steps in patient care and the events when the nurse has to contact the treating rheumatologist. Generally, the nurse operates under the supervision of the rheumatologist.

#### Control intervention: standard care

Patients in the control group will be treated by their rheumatologist according to the standards of care in rheumatology, e.g. the treat-to-target principle. Usually, standard care is assessed every 3 months, although additional appointments may be necessary according to the disease course.

### Primary outcomes

There are two primary outcomes, which will be tested hierarchically: disease activity and health-related quality of life.

Disease activity is probably the most important patient-related outcome in chronic inflammatory arthritis. Thus, the mean change in disease activity in the course of 1 year was chosen as the first of the two primary outcomes. Disease activity is assessed using the well-established DAS28 [7]. DAS28 is a compound score comprising the number of tender joints (out of 28), the number of swollen joints (out of 28), the erythrocyte sedimentation rate, and the patient’s assessment of disease activity. The range of the score is 0 (no activity) to 10 (maximum disease activity).

We expect the intervention group to be superior with regard to changes in health-related quality of life without a relevant difference in disease activity after 1 year. Health-related quality of life will be assessed by the EQ-5D-5L [8], which has five dimensions: (1) mobility, (2) self-care, (3) usual activities, (4) pain and discomfort, and (5) anxiety and depression. Each dimension has five levels: (1) no problems, (2) slight problems, (3) moderate problems, (4) severe problems, and (5) extreme problems. This five-level scoring system has increased sensitivity and a reduced ceiling effect, amongst other advantages, compared to the three-level scoring system. For details on scoring and evaluation, see [8].

### Secondary outcomes

Secondary outcomes include other established assessments in rheumatology, as well as evaluations of organizational issues, patient satisfaction with medical treatment, and resource consumption.

One of the most important prognostic markers in rheumatoid arthritis is physical functioning, which will be measured by the validated Hannover Physical Functioning Questionnaire (FFbH) [9]. This assessment is comparable to the internationally applied Health Assessment Questionnaire (HAQ) [10]. Patients are asked, if they are able to perform 18 activities of daily living. They answer on a three-point scale (“yes,” “yes, but with difficulty,” and “no or only with assistance”). The patient’s physical activity will be assessed via the PRISCUS Physical Activity Questionnaire (PAQ) [11], which has ten items that assess the time spent in domestic activities (e.g., housework), sporting activities (e.g., riding a bicycle), and inactivity (e.g., sedentary activity) during the prior week. The tendency to depressive moods is assessed by the two-item Patient Health Questionnaire (PHQ-2) [12]. Further patient-related outcomes are the patient’s estimate of (1) disease activity (numeric rating scale 0–10 [NRS]), (2) pain (NRS), (3) fatigue (NRS), (4) sleep disturbances (NRS), and (5) duration of morning stiffness. The C-reactive protein (CRP) is a standard feature of laboratory analyses, and it will be assessed in the course of the intervention. Smoking status may influence the course of chronic inflammatory diseases and will be subject of patient education by the nurse. Therefore, it will be assessed as a secondary outcome.

In addition, patients will be asked about how long they need to wait for an appointment in their practice, the availability of the rheumatologist or the nurse for inquiries, their satisfaction with the information provided, their satisfaction with their relationship with the rheumatologist or the nurse, and their satisfaction with the coordination and communication done by the different professional groups.

These outcomes are compared to direct and indirect costs and other resource consumption (e.g. duration and costs of nurse-led care vs. standard care). A EULAR recommendation [4] is “to perform cost-effectiveness studies across different European countries, on the role of the nurse in basic and advanced practice.” There are already British [5] and Dutch [13] but no German studies on the costs and efficiency of nurse-led care in rheumatology. Within this study, an economic evaluation will be done using the Questionnaire for Health-Related Resource Use in an Elderly Population (FIMA) [14]. The components of FIMA are as follows: (1) outpatient medical care, (2) remedies, (3) nursing and domestic care, (4) semi-residential care (daycare) and short-term care, (5) benefits of statutory nursing-care insurance, (6) medication, (7) rehabilitation measures, (8) (partly) inpatient hospitalization, (9) medical aids, (10) relocation, (11) type of housing, and (12) periods of incapacity for work.

### Other trial data

Baseline data for patient characterization comprise sociodemographic data (age, gender, academic qualifications, professional qualifications, occupation, and retirement status) and data on comorbidities. Newly diagnosed diseases will be continuously assessed within the course of the intervention.

CDAI [15] will be assessed at each visit. The score consists of the number of swollen joints, the number of tender joints, the patient’s global assessment of disease activity, and the evaluator’s global assessment (this will be either the rheumatologist or the nurse depending on group allocation in this study). CDAI will be used for participants with either rheumatoid arthritis or psoriatic arthritis (polyarticular manifestation). The score will be used to adjust patient treatment if necessary. Finally, the body surface area [16] is an established way to assess the percentage of skin affected by psoriatic arthritis.

### Assignment of interventions

Patients who are correctly enrolled via screening fax and have completed a telephone interview will be randomized by the data management center to either standard care or nurse-led care. Randomization will be performed using blocks with a variable block length stratified by center. The allocation sequence is accessible only to members of the data management team. Study centers will be informed by fax of the group allocation of each patient before their baseline visit.

In this study, trial participants and care providers cannot be blinded to group allocation. The outcome assessors will be blinded. Patients are asked not to tell the assessor or the call center agents to which group they are assigned. Data analysts are also blinded.

### Data collection

**Fig. 1:**
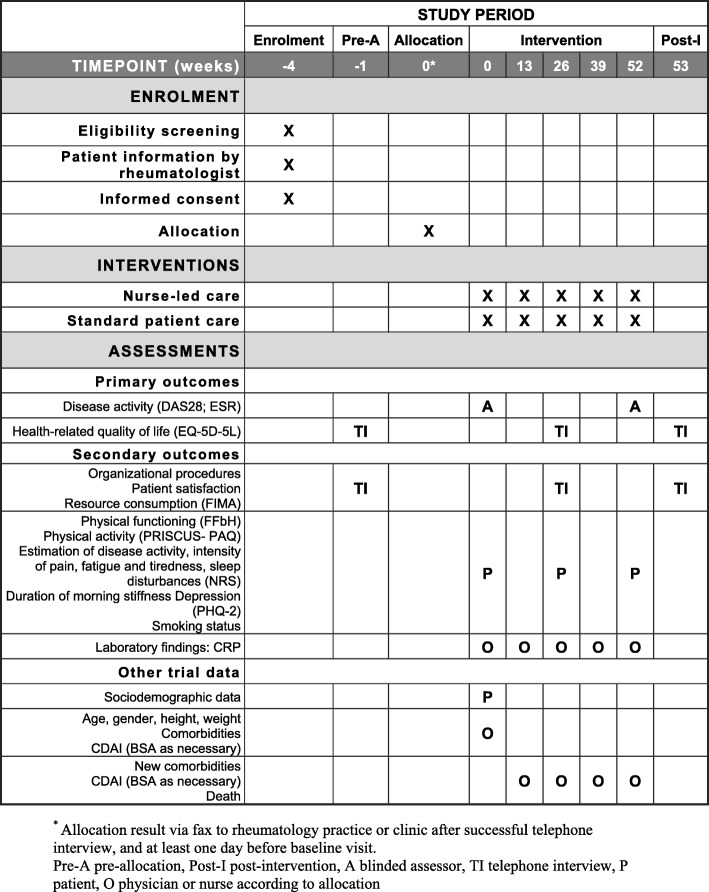
Schedule of enrolment, interventions, and assessments. A blinded assessor, BSA body surface area, CDAI Clinical Disease Activity Index, CRP C-reactive protein, DAS28 28-joint Disease Activity Score, EQ-5D-5L Five-dimension Five-level Quality of Life, ESR erythrocyte sedimentation rate, FFbH Hannover Physical Functioning Questionnaire, FIMA Questionnaire for Health-Related Resource Use in an Elderly Population, NRS numeric rating scale, O physician or nurse according to allocation, P patient, PAQ Physical Activity Questionnaire, PHQ-2 Two-item Patient Health Questionnaire, TI telephone interview

Study data will be collected at baseline after randomization, and after 13, 26, 39, and 52 weeks. Figure 1 is the schedule of enrolment, interventions, and assessments.

At the beginning and at the end of the intervention for a participant, the first primary outcome, DAS28, will be assessed by a blinded external assessor in the practice or outpatient clinic. The assessors are qualified assistant doctors from the Herne Rheumatology Center. They are trained in performing standardized assessments using DAS28 and have received a written copy of the standard operating procedures.

The other primary outcome, health-related quality of life, and secondary outcomes, including organizational procedures, patient satisfaction, and resource consumption, will be assessed via a computer-assisted standardized telephone interview at baseline as well as after 26 and 52 weeks.

The patient will be asked to complete questionnaires at baseline, after 6 months, and at the end of the intervention. Each questionnaire asks about secondary outcomes, such as smoking status, physical functioning, physical activity, depression, estimation of disease activity, intensity of pain, fatigue, tiredness, sleep disturbances, and duration of morning stiffness. The patient additionally provides sociodemographic information at baseline.

The rheumatologist or the nurse will document the participant’s age, gender, height, and weight at baseline. Furthermore, they will assess the CRP as a secondary outcome, (new) comorbidities, the CDAI (and body surface area as necessary), or death of the patient at each routine control examination every 3 months.

### Data management

The Department of Medical Informatics, Biometry and Epidemiology (AMIB) at Ruhr-University Bochum will manage the data and undertake the statistical analysis. After each study visit, the study centers will send the relevant part of the patient’s case record via fax to the data management center. Practices may be asked in writing for corrections or additional information, as necessary.

All study data will be collected in strictly pseudonymous form. Patients may participate in the trial only if they sign the privacy statement and consent to their data being transferred for analysis. Only authorized personnel will make data entries in and corrections to case record forms. In addition to participating practitioners and rheumatology nurses, specifically trained medical staff may also assist in recording data.

Data processing will start after the last participant has had their final telephone interview and after data clearing. Double data entry, data verification, data coding, and consistency checks are standard data management processes that will be used to maximize data quality.

AMIB will store all study documents for at least 10 years. Thus, study results can be reproduced after the end of the funding period. Case record forms and written informed consent forms will also be stored for at least 10 years in the relevant rheumatology practice or clinic.

### Statistical analysis

This trial has a two-part primary endpoint that will be hierarchically tested. Non-inferiority of the intervention group compared to the standard care group will first be examined regarding the difference in disease activity (DAS28) after 1 year. A *t*-test of non-inferiority (one-sided, α = 0.025, non-inferiority-margin 0.4 in the DAS28 score) will be performed. The analysis population consists of those who completed the study per protocol.

If nurse-led care is found to be non-inferior, its superiority over standard care will be tested with regard to health-related quality of life (EQ-5D-5L). This analysis will be performed with all randomized patients based on the intention-to-treat principle using multiple imputation.

The analysis of the secondary outcomes will use all available data for the per protocol population. Continuous data will be compared for the two groups using an analysis of covariance adjusting for baseline data. Analyses of categorical variables or proportions will be performed using a chi-squared test. An additional repeated-measures analysis will be done for all quantitative endpoints taken at different time points. Details of the primary, secondary, and all additional analyses are documented in the statistical analysis plan.

### Evaluation of delegation

The delegation of a physician’s tasks to a specialized rheumatology nurse will be evaluated as follows:

1. Quality of the specialization course: All participating nurses will evaluate the training. In addition, nurses will be assessed by the teacher during the course. At the end of the course, each nurse will be evaluated by comparing her joint examination to that done by an assessor for the same patient. The assessor will be a physician and the nurse will not be told of the assessor’s results.
2. Quality of the results: Whether delegation is successful will be apparent in the primary and secondary outcomes. The analysis will determine whether nurse-led care is as effective as standard care. This will be reflected in clinical outcomes such as the course of the disease but also in subjective outcomes such as patient satisfaction with the new form of health care.

### Monitoring

The Coordinating Center for Clinical Trials at the Philipps University of Marburg is responsible for monitoring each participating study center based on its standard operating procedures for monitoring. It is independent of the trial sponsor and there are no competing interests. Audit visits will ensure that sites adhere to the study protocol. Data collection forms will be checked for completeness and plausibility. The monitoring staff will have direct access to source data (original medical records) during their visits to verify the existence of participants and to check the inclusion and exclusion criteria as well as written informed consent forms. The findings from each audit will be summarized in a report. Three audits for each participating study center are planned.

Due to its experience, the Coordinating Center for Clinical Trials will additionally support the coordinating center and the data management team at the University of Bochum in an advisory capacity regarding data monitoring and safety aspects, and will provide general trial oversight.

## Discussion

The expected improvement in rheumatology care is important. It should give patients better access to the rheumatology team. The enhanced participation of the specialized rheumatology nurses in providing comprehensive patient care may improve the endpoints of disease activity and health-related quality of life. The optimization of the treat-to-target concept should result in more patients reaching their target range (disease activity low or in remission) with a better disease progression in the mid-term. Outcomes relevant to patients, such as quality of life, functional capacity, earning capacity, and participation, may also reflect the improvement. Additionally, we assume that the benefits of nurse-led patient care will significantly outweigh the implementation costs. Finally, the project may form a basis for the sustainable implementation of nurse-led care as standard practice in Germany.

### Trial status

The project started in September 2017, and the nurses were trained in June and August 2018. Patient recruitment started in September 2018 and ended in August 2019. The data analysis will start one year later once the final participant has completed the intervention. The expected end date of the project is in September 2020.

## Supplementary information

**Additional file 1.** SPIRIT 2013 Checklist: Recommended items to address in a clinical trial protocol and related documents.

## Data Availability

Data sharing is not applicable to this article as it is a study protocol without data analysis.

## Abbreviations

BSA: Body surface area
CDAI: Clinical Disease Activity Index
CRP: C-reactive protein
DAS28: 28-joint Disease Activity Score
EQ-5D-5L: Five-dimension Five-level Quality of Life
ESR: Erythrocyte sedimentation rate
EULAR: European League Against Rheumatism
FFbH: Hannover Physical Functioning Questionnaire
FIMA: Questionnaire for Health-Related Resource Use in an Elderly Population
NRS: Numeric rating scale
PAQ: Physical Activity Questionnaire
PHQ-2: Two-item Patient Health Questionnaire
